# Study on the Mechanical Properties, Wear Resistance and Microstructure of Hybrid Fiber-Reinforced Mortar Containing High Volume of Industrial Solid Waste Mineral Admixture

**DOI:** 10.3390/ma15113964

**Published:** 2022-06-02

**Authors:** Hao Wu, Yanmin Jia, Zhu Yuan, Zhijia Li, Tao Sun, Jiahao Zhang

**Affiliations:** School of Civil Engineering, Northeast Forestry University, Harbin 150040, China; wuhao19@nefu.edu.cn (H.W.); zhu_yuan163@163.com (Z.Y.); lizhijia@nefu.edu.cn (Z.L.); suntao@nefu.edu.cn (T.S.); 2019213537@nefu.edu.cn (J.Z.)

**Keywords:** hybrid fiber-reinforced mortar, industrial solid waste, mineral admixtures, mechanical properties, wear resistance, microstructure

## Abstract

The use of a high volume of industrial solid waste mineral admixture and hybrid fiber can greatly reduce the amount of cement in mortar or concrete, improve its performance, ensure the service properties of mortar or concrete, and reuse industrial solid waste to reduce the environmental burden, which has significant research significance. In this paper, the mechanical properties, wear resistance and microstructure of hybrid fiber-reinforced mortar (HFRM) with a high content of industrial solid waste mineral admixture were systematically studied under different water/binder ratios. Mineral admixtures include fly ash, silica fume and granulated blast furnace slag (slag). The total content of hybrid glass fiber (GF) and polypropylene fiber (PPF) was 2% by volume fractions, and six different water/binder ratios ranging from 0.27 to 0.62 were used. The following conclusions were drawn: fibers have a significant negative effect on the properties of mortars with a low water/binder ratio (w/b = 0.27) and high content of mineral admixtures. In general, the effect of adding hybrid fiber on improving the wear resistance of mortar is more obvious. The average residual weight of hybrid fiber-reinforced mortar is the highest after the wear resistance test. Comprehensively considering the compressive strength, flexural strength, wear resistance and microstructure of the mortar samples, G8PP2-0.40 is the optimal mix ratio. At this time, the replacement rates of fly ash, silica fume and slag are: 20%, 5% and 30%, the water/binder ratio is 0.40, and the content of GF and PPF is 1.6% and 0.4%, respectively.

## 1. Introduction

Infrastructure construction around the world uses a lot of mortar and concrete, which consumes a lot of common Portland cement, and it is well known that the production of cement is one of the major contributors to carbon dioxide emissions, affecting climate change and global warming. This environmental issue is likely to become more severe as demand for cement increases [1,2]. Cement consumption can be reduced by adding natural or artificial mineral admixtures to mortar or concrete mixtures [3,4,5,6,7,8,9,10]. The mineral admixture is mainly composed of one or more oxides such as silicon, calcium, and aluminum, and it has a specified fineness. According to the source, mineral admixtures can be divided into three categories: natural, artificial and industrial solid waste. Natural mineral admixtures include volcanic ash, tuff, zeolite powder, siliceous shale, etc.; artificial mineral admixtures include calcined shale, metakaolin, etc.; industrial solid waste mineral admixtures include granulated blast furnace slag, fly ash, silica fume, etc. [11,12,13]. Replacing part of cement in mortar or concrete with mineral admixtures can reduce cement consumption, thereby reducing carbon dioxide emissions and natural resource consumption [4,5]. At the same time, mineral admixtures can also improve the performance or strength of concrete mixture [11,14,15,16]. In addition, the accumulation of industrial solid waste such as fly ash will occupy many land resources, and dust will also cause serious air pollution. Therefore, replacing cement with industrial waste mineral admixtures is the secondary utilization of industrial solid waste, which has significant ecological and environmental benefits [17,18,19,20].

Among the mineral admixtures of industrial solid waste, fly ash is a by-product of thermal power generation and belongs to aluminosilicate glassy pozzolanic active materials. Studies have shown that fly ash significantly affects the microstructure and mechanical properties of hydrated calcium gels. Fly ash reduces the weight percentage of calcium hydroxide in the hydration product, but it increases the grain size, indentation modulus and hardness of calcium hydroxide [5]. Due to the refinement of the pore structure caused by the pozzolanic reaction of fly ash, the permeability of the cement slurries incorporating fly ash is lower than that of pure Portland cement slurries at the curing age of 180 days [21]. Therefore, the cement slurry mixed with fly ash has better mechanical properties and durability [21,22,23]. Silica fume is a powder material with amorphous silica as the main component obtained by collecting the flue dust discharged from the smelting of ferrosilicon alloy or industrial bulk silicon. As an ideal admixture for improving the performance of concrete, silica fume acts as a mineral admixture for high-performance concrete, creating more discontinuous and impermeable pore structures in concrete. The reduced high permeability of silica fume is due to the refinement of pore size and matrix densification, the reduction of Ca(OH)_2_ content, and the refinement of the cement paste–aggregate interface. During the hydration process, the pozzolanic reaction between silica fume and calcium hydroxide gradually densifies the transition interface [24,25]. Mixing fly ash and silica fume can increase the setting time, slow down the hydration rate, reduce the hydration heat and temperature, and suppress shrinkage [6]. The application of silica fume in fly ash concrete can significantly improve the early fracture toughness of these materials [26]. Slag is a by-product of pig iron smelting and is suitable as an active admixture in cement or as a mineral admixture for concrete. The use of slag in cement-based materials is increasing because it performs well in marine and other corrosive environments [27,28], and it can achieve mechanical properties similar to or better than ordinary cement mortars [29,30]. For the case where two or three kinds of mineral admixtures are added to the concrete mixture, it is believed that the addition of fly ash and slag can improve the compressive strength and fracture performance of concrete in the environment of low humidity and large temperature change. Replacing cement with 10% fly ash and 20% slag can significantly improve the compressive strength, initial fracture toughness, unstable fracture toughness and fracture energy compared with ordinary concrete. By analyzing the concrete mesostructure, the incorporation of fly ash and slag can attenuate the adverse effects of low humidity and large temperature changes on the concrete mesostructure and cement hydration [31]. The ternary blend system of silica fume, slag and cement can obtain relatively high compressive strength [32].

Fiber-reinforced mortar or fiber-reinforced concrete is a new composite material developed rapidly in recent years, with excellent crack resistance, bending resistance, wear resistance and other properties. Incorporating chopped and uniformly distributed fibers into the mortar or concrete not only has the excellent characteristics of the mortar and concrete itself, at the same time, fibers limit the development of cracks in mortar and concrete, so that the original brittle concrete material exhibits high toughness and ductility, as well as excellent wear resistance and other characteristics [11]. Fiber-reinforced mortar or concrete has been widely used in the field of civil engineering because of its good physical and mechanical properties. However, a single type of fiber-reinforced concrete generally only has superior properties in certain specific aspects, which may lead to certain limitations in its popularization and application. Hybrid fiber-reinforced mortar or concrete mixed with two or more fiber types can simultaneously have excellent comprehensive properties such as strength, toughness and durability due to the multiple reinforcing effects of fibers [33,34,35,36,37,38,39].

Regarding the effect of mineral admixtures on the properties of fiber-reinforced cement paste, mortar or concrete, in a rheological study, concrete mixtures with mineral admixtures have better fresh concrete properties. Steel fibers increase the flexural strength of concrete and prevent it from brittle failure [40]. In the study of the effect of mineral admixtures on the properties of ultra-high performance fiber-reinforced concrete in the fresh state, it was found that due to factors such as the difference in particle shape and the strength of inter-particle bonds, the addition of metakaolin and silica fume on the plastic viscosity of fresh mixtures will have different effects. The regularity and roundness of silica fume particles are conducive to fluidity, the flat shape of the metakaolin particles is the main factor responsible for the high plastic viscosity values and the large increase in plastic viscosity with time in the fresh mixture [41]. Studies show that fly ash and silica fume can significantly prolong the aging effect of basalt fiber on the flexural strength enhancement of cement mortar. Microstructural analysis shows that the content of calcium hydroxide crystals in the cement matrix and the corrosion degree of basalt fibers are reduced after the addition of fly ash and silica fume, and the interface properties between the basalt fibers and the cement matrix were improved [42]. By using the accelerated aging method of 80 °C moist heat curing, the durability of GF in cement-based composites was investigated, and it was found that the time-dependent change of the mechanical properties of glass fiber-reinforced Portland cement is the result of the combined effect of the increase in cement paste strength and the corrosion damage of GF. With the prolongation of curing time, fly ash keeps the cement paste strength increasing, and on the other hand, it inhibits the corrosion of GF. As a result, the corrosion of GF was reduced, and the flexural strength kept increasing [43]. The durability and ductility of ordinary cement concrete can be improved by incorporating fiber and mineral admixtures at the same time. Incorporating silica fume increases the efficiency of the fibers against tensile and flexural loads. The composite effect of fibers and silica fume is more favorable than the sum of the effects of the two alone. Silica fume helps to reduce the water content and chloride ion penetration of fiber-reinforced concrete, thereby improving the acid resistance of them [44]. The mechanical properties and durability of recycled aggregate concrete can be significantly improved by the combination of mineral admixtures and hook-end steel fiber. The benefits of the combined addition of hook-end steel fibers and mineral admixtures are significantly higher than the sum of the benefits obtained by combining hook-end steel fibers and mineral admixtures individually [45]. The study on the mechanical properties of glass fiber-reinforced cement mixed with fly ash and slag indicates that fly ash and slag may have the ability to inhibit the chemical corrosion of GF in glass fiber-reinforced cement [46].

The water/binder ratio is an important parameter in the mix ratio design of cement-based material, which has a significant influence on the workability, setting time, mechanical properties and durability of mortar or concrete. Studies also show that the water/binder ratio can affect the optimal dosage of fiber added into the fiber-reinforced concrete [33]. Therefore, when studying the influence of mineral admixtures and fibers on concrete composites, the water/binder ratio is a factor that cannot be ignored.

The above analysis shows that there have been extensive studies on the influence of mineral admixture, single type fiber and hybrid fiber on cement paste, mortar or concrete, and certain results have been achieved. However, there are a few reports that simultaneously study the effects of industrial solid waste mineral admixtures (especially high-content mineral admixtures), hybrid fiber and water/binder ratio on the properties of cement-based materials. The use of high content of industrial solid waste means that the amount of cement used can be significantly reduced, thus reducing the environmental burden. However, adding too much mineral admixture may reduce some properties, such as mechanical properties or wear resistance, to a certain extent. Chopped fibers, especially hybrid fibers, have the potential to improve these properties. Therefore, the simultaneous study of high content mineral admixtures and hybrid fibers is of great significance to solve engineering problems in a practical application. The amount of water is an important factor in the mix design of cementitious composite. In this study, water is also an indispensable factor in studying the optimal dosage of mineral admixtures and hybrid fibers and fully exerting the synergistic effect of mineral admixtures and hybrid fibers. Therefore, in this study, fly ash, slag and silica fume as industrial solid wastes were selected as mineral admixtures to partially replace cement, with the highest replacement rate reaching 85%. The short-cut GF and PPF were selected as micro-reinforced materials, and six different water/binder ratios ranging from 0.27 to 0.62 were set. The effects of industrial solid waste mineral admixtures, hybrid fibers and water/binder ratio on the properties of cement mortar, including compressive strength, flexural strength, wear resistance and microstructure, were comprehensively studied. The mechanism of action of the above materials on the properties of cement mortar was analyzed in depth. The optimal mixing ratio of HFRM containing high content mineral admixtures was selected to provide a reference for theoretical research and engineering applications.

## 2. Materials and Methods

### 2.1. Materials

#### 2.1.1. Ordinary Portland Cement

The P.O 42.5 ordinary Portland cement was used in this study, and its physical and mechanical properties are shown in Table 1. The testing standards for the physical and mechanical properties of cement are as follows. The soundness and setting time test procedure was conducted according to the standard GB/T 1346-2011 [47]. The specific surface area test procedure was conducted according to the standard GB/T 8074-2008 [48]. The strength test procedure was conducted according to the standard GB/T GB/T 17671-1999 [49]. The chemical compositions of cement are shown in Table 2.

#### 2.1.2. Fly Ash

The fly ash is Class I fly ash produced locally in Harbin, China. The chemical compositions of fly ash are shown in Table 3.

#### 2.1.3. Silica Fume

The chemical compositions of silica fume are shown in Table 4.

#### 2.1.4. Slag

The strength grade of the slag is S95, and the density is 2.89g/cm^3^.The chemical compositions of slag are shown in Table 5.

#### 2.1.5. Fibers

**Figure 1:**
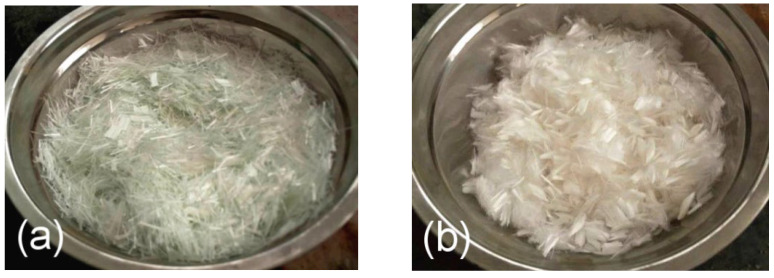
Appearance of the used fibers: (**a**) GF and (**b**) PPF.

The fibers used in the test are chopped GF and PPF with a length of 12 mm. The morphology and main parameters of the fibers used are shown in Figure 1 and Table 6.

#### 2.1.6. Fine Aggregate

Natural river sand was selected as fine aggregate and its fineness modulus Mx = 2.66.

#### 2.1.7. Water

The water used in the test is the municipal water supply for the city.

### 2.2. Mix Proportions

Because the influence of mineral admixture, water/binder ratio and fiber content on mortar properties is considered simultaneously, there are too many variables to be controlled. Therefore, this study combined the orthogonal experimental approach to carry out the mixed design. The orthogonal experimental approach refers to an experimental design method that studies multiple factors and multiple levels. When there are 3 or more factors involved in the experiment, and there may be interactions among the factors, the workload of the experiment will become very large and even difficult to implement. In response to this problem, orthogonal experimental design is undoubtedly a better choice. According to the orthogonal experimental approach, the experimenter can select representative points from the comprehensive test to carry out the test and can achieve the equivalent results of a large number of comprehensive tests with a minimum number of tests [50].

The selection of orthogonal test tables should be based on the specific factors and levels of the test. In this experiment, the following six factors were included. Firstly, there were two key factors affecting the working performance and mechanical properties of the material: the total amount of binder and the water/binder ratio. Secondly, three kinds of industrial solid wastes were included: fly ash, silica fume and slag. Thirdly, the fiber content was selected.

The use of fly ash in concrete manufacturing could form significant energy and environmental benefits. In addition, the use of fly ash as a partial replacement for cement reduced the water/binder ratio and the heat of hydration from the pozzolanic reaction, and it improved the strength and durability of mortar or concrete, especially the long-term compressive strength. According to references [16,21,40,51,52,53,54], the maximum content of fly ash in this study was 50% of the total binder. Slag could undergo a secondary reaction with Ca(OH)_2_ produced by cement hydration, which promoted the hydration degree of cement, improved the pore structure and strength properties, and increased durability [16,45,55]. According to the above literature, the slag dosage in this study was set at 15% and 30%. As a high pozzolanic active material, silica fume had very small particle size and was mostly spherical, which resulted in a strong filling effect between silica fume and powder materials such as cement, fly ash and slag. Silica fume could make concrete structures more dense, reduce porosity, and refine the pore structure, thereby significantly improving concrete strength and durability. When preparing concrete, silica fume and other mineral admixtures such as fly ash and slag were usually “double-mixed” or “triple-mixed”, which could improve the strength and durability and control the production cost of concrete materials [40,45,52]. Considering the improvement effect and production cost, the silica fume content in this study was 0% and 5%. According to references [37,41,56,57], the total content of hybrid fibers was set to 2%. According to the standard JGJ/T 98-2010 [58], the total binder and the water/binder ratio were set with reference to the mortar with a strength grade not lower than M15.

The ranges and levels of the above factors were set as shown in Table 7. It can be seen that the number of levels of each factor in this study is not exactly equal, which is also called a mixed-level multi-factor problem. The orthogonal arrays of the mixed ratio were obtained by using the tool of the data science algorithm platform. It has been proved that this orthogonal array has the characteristics of uniform dispersion and uniformity and comparability. The orthogonal arrays were divided into 4 groups according to the fiber incorporation method. Each group was rearranged according to the order of the increase in the water/binder ratio. The mix proportions of mortar mixes are shown in Table 8.

### 2.3. Specimen Preparation and Curing

The cement, mineral admixture, fine aggregate, fiber and water are put into the cement mortar mixing pot and mixed evenly and then put into the mold. After vibrating on the shaking table, they are maintained in the laboratory for 24 h; then, they are demolded and maintained to the specified curing age under standard conditions.

### 2.4. Test Procedure

The strength test procedure was conducted according to the standard GB/T 17671-2021 [49]. The mortar sample used for the mechanical property test is a prism with a size of 40 mm × 40 mm × 160 mm, 3 samples for each mixed ratio are subjected to the flexural strength test, and the 6-segment samples after the flexural strength test are completed for the compression test. The size of the mortar sample used for the wear resistance test is 50 mm × 50 mm × 50 mm cube. The size of the mortar sample used for microstructure testing is 40 mm × 40 mm × 160 mm prism. A Quanta 200 scanning electron microscope (made by FEI company, Eindhoven, Netherlands) was used to observe the micromorphology of the mortar specimens. The SEM test procedure was conducted according to the standard ASTM C1723-2010 [59]. The sampling method, sampling principle and microstructure observation test process are the same as in reference [33,60].

## 3. Results and Discussion

### 3.1. Compressive Strength

**Figure 2:**
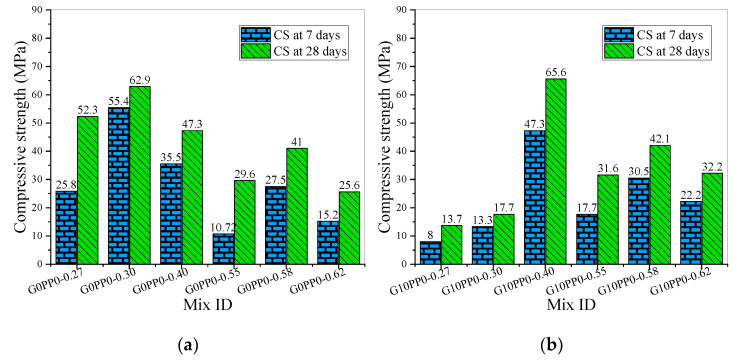

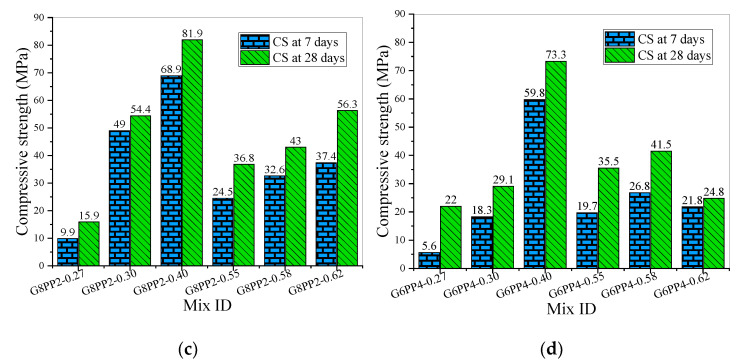
Compressive strength of mortar: (**a**) The compressive strength of mortar in group 1; (**b**) The compressive strength of mortar in group 2; (**c**) The compressive strength of mortar in group 3; (**d**) The compressive strength of mortar in group 4.

Figure 2 shows the results of the compressive strength test of the mortar specimens in this study. Figure 2a shows the test results of the first group of samples. It can be seen that when the total fiber content is 0, G0PP0-0.30 has the highest compressive strength, and the strength values at 7 days and 28 days curing age are 55.4 MPa and 62.9 MPa, respectively. The total amount of binder in this mortar sample is 400 kg/m^3^, which is not the highest amount, among which fly ash, silica fume and slag account for 40%, 5% and 15%, respectively. The 7-day compressive strength of G0PP0-0.55 is 10.72 MPa, which is only 36.22% of the 28-day strength. That is, the growth rate of the compressive strength of G0PP0-0.55 in the first 7 days is far lower than the normal level. This is because the replacement ratio of cement by mineral admixtures in this sample reached 85%, especially the replacement ratio of fly ash reached 50%. At room temperature and without adding alkali activator, the hydration reaction rate of fly ash is much lower than that of cement. When the fly ash content increases, the Ca(OH)_2_ in the binary fly ash cement binary system decreases significantly [51]. Since the content of Portland cement in G0PP0-0.55 is only 15%, the reduction of Ca(OH)_2_ caused by the hydration of Portland cement also delays the pozzolanic reaction of fly ash [52]. Therefore, the excessively high replacement rate of mineral admixtures, especially fly ash, resulted in the lowest 7-day compressive strength. This also explains why the amount of binder of G0PP0-0.55 is 480 kg/m^3^, the largest amount in group 1, but the 28-day strength is only 29.6 MPa, which is only 47.06% of that of G0PP0-0.30. This result is consistent with reference [52]; that is, the compressive strength decreases with the increase in fly ash content.

Figure 2b shows the compressive strength of the specimens with 2% GF and 0% PPF. It can be seen that the 7-day and 28-day compressive strength of G10PP0-0.40 is 47.3 MPa and 65.6 MPa, respectively, which is much higher than the strength of other samples in group 2, and the compressive strength of G10PP0-0.27 is the lowest. The 7-day and 28-day compressive strengths of G10PP0-0.27 are 8 MPa and 13.7 MPa, respectively, which are only 16.91% and 20.88% of that of G10PP0-0.40. The reasons are analyzed as follows. Firstly, the binder dosage of G10PP0-0.27 is 350 kg/m^3^, which is lower than that of G10PP0-0.40 (400 kg/m^3^). Secondly, under the same substitution rate of silica fume and slag, the fly ash replacement ratio of G10PP0-0.27 is 40%, which is higher than that of G10PP0-0.40. Thirdly, the water/binder ratio of G10PP0-0.27 is only 0.27; adding 2% GF will significantly reduce the workability of the fresh mortar, making it difficult to mold the fresh mortar and increasing the structural defects such as pores in the mortar.

In Figure 2c, the content of GF and PPF are 1.6% and 0.4% by volume fraction, respectively. It can be seen that in group 3, G8PP2-0.40 has the highest compressive strength value. The 7-day and 28-day compressive strengths are 68.9 MPa and 81.9 MPa, respectively. Through comparison and analysis, it can be seen that the binder amount of G8PP2-0.40 is 350 kg/m^3^, which was the lowest in Group 3. However, due to the reasonable mineral admixtures replacement ratio and water/binder ratio, as well as the promiscuous effect of the hybrid fibers, the strength value of G8PP2-0.40 is optimal. Since the slag content of G8PP2-0.40 is 30%, the hydration heat of slag is higher than that of fly ash, and the reaction temperature increases, which promotes the hydration of binder and improves the reaction degree of slag and fly ash [55]. In addition, the use of 5% silica fume resulted in improved compressive strength of the samples due to the composition of silica fume being a highly amorphous silica phase, leading to a more efficient pozzolanic reaction [52]. The high modulus and high strength of GF can effectively improve the strength of mortar, but the inherent brittle behavior of GF cannot improve the ductility of mortar. PPF is more effective in improving ductility and reducing cracking as a type of low-strength fiber. In general, adding hybrid fibers to concrete can comprehensively improve many of its engineering properties, such as toughness, ductility, energy absorption, and durability, compared to a single type of fiber [34,56]. The hybrid fibers in G8PP2-0.40 benefit from each individual fiber and exhibit a synergistic effect. The compressive strength values of G8PP2-0.27 are the lowest at 9.9 MPa and 15.9 MPa, which are only 14.37% and 19.41% of the corresponding strength values of G8PP2-0.40. The reasons are analyzed as follows. Firstly, the replacement ratio of mineral admixtures is too high, reaching 85%, and the replacement ratio of fly ash has reached 50%. Secondly, the water/binder ratio is only 0.27, which means too little water is used.

The content of GF and PPF in the mortar samples of Figure 2d is 1.2% and 0.8%, respectively. The samples with the minimum and maximum strength values are G6PP4-0.27 and G6PP4-0.40, respectively. The 7-day and 28-day compressive strength values of G6PP4-0.27 are 5.6 MPa and 22 MPa, respectively. This is because G6PP4-0.27 also has the lowest water/binder ratio (0.27) and the minimum binder amount (only 300 kg/m^3^); after adding 2% hybrid fiber, the binder wrapped around the fibers is seriously insufficient to play the role of fiber micro-reinforcing material. In reference [56], when the water/binder ratio of mortar is 0.43 and the total content of hybrid steel fiber, palm fiber and synthetic fiber is 2%, the 28-day compressive strength ranges from 52.8 to 59.3 MPa, which is far lower than the strength value of HFRM (G8PP2-0.40 and G6PP4-0.40) with similar water/binder ratio in this study. One of the reasons may be that the mineral admixtures of G8PP2-0.40 and G6PP4-0.40 contain not only silica fume but also fly ash and slag [56].

To sum up, G8PP2-0.40 is the optimal mix proportion. In this case, the replacement ratios of fly ash, silica fume and slag are 20%, 5% and 30%, respectively, the water/binder ratio is 0.40, and the GF and PPF content are 1.6% and 0.4% by volume fractions, respectively.

### 3.2. Flexural Strength

**Figure 3:**
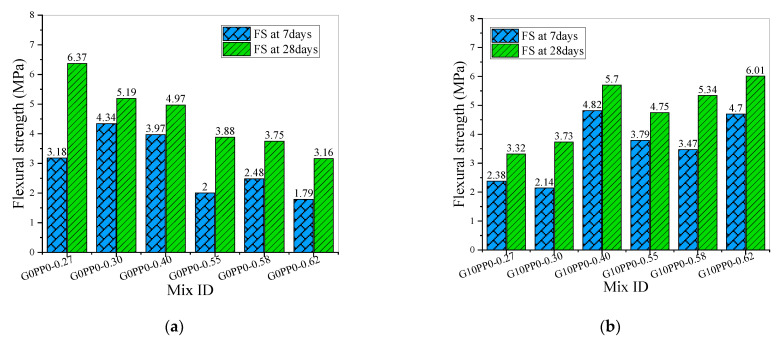

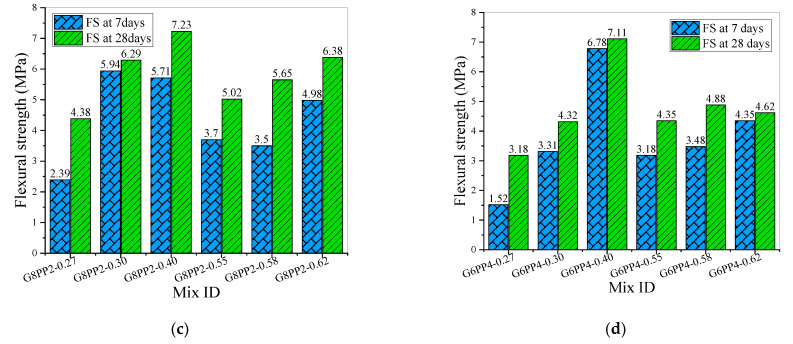
Flexural strength of mortar: (**a**) The flexural strength of mortar in group 1; (**b**) The flexural strength of mortar in group 2; (**c**) The flexural strength of mortar in group 3; (**d**) The flexural strength of mortar in group 4.

Figure 3 shows the flexural strength test results of the HFRM with high volume mineral admixture. It can be seen that in Figure 3a, without adding fibers, the 7-day flexural strength of mortar samples has exactly the same change trend as the corresponding 7-day compressive strength in Figure 2a. However, the 7-day flexural strength of G0PP0-0.30 is only 36.48% higher than that of G0PP0-0.27, and the 7-day compressive strength of G0PP0-0.30 is 114.73% higher than that of G0PP0-0.27, while the 28-day flexural strength and compressive strength of G0PP0-0.27 increased by nearly 100% compared with the 7-day strength value. Therefore, in the samples without fiber, the flexural strength of GOPPO-0.27 is optimal, which reached 6.37 MPa.

In Figure 3b, the flexural strength of G10PP0-0.27, G10PP0-0.30 and G10PP0-0.40 has a similar change trend to the compressive strength. G10PP0-0.40 has the largest flexural strength, and the 28-day flexural strength is 5.7 MPa. In the samples of G10PP0-0.55, G10PP0-0.58 and G10PP0-0.62, compared with the compressive strength, the flexural strength of G10PP0-0.62 was not decreased, but it significantly increased and reached 6.01 MPa at 28 days, which was the maximum strength in group 2.

In Figure 3c,d, the variation trend of the mortar sample’s flexural strength was completely consistent with that of the corresponding compressive strength in Figure 2c,d. The 28-day flexural strength of G8PP2-0.40 reached 7.23 MPa. In Section 3.1, the comparative analysis shows that the compressive strengths of G8PP2-0.40 and G6PP4-0.40 are higher than all the 28-day compressive strengths of reference [56], but the flexural strength in this study is lower than that of reference [56]. This is mainly because the steel fiber content of test samples in reference [56] is between 1% and 2%, and this content of steel fiber has a good effect on improving the flexural strength of the samples. Considering the flexural strength test results in Figure 3, G8PP2-0.40 was also the optimal mix proportion.

### 3.3. Wear Resistance

**Figure 4:**
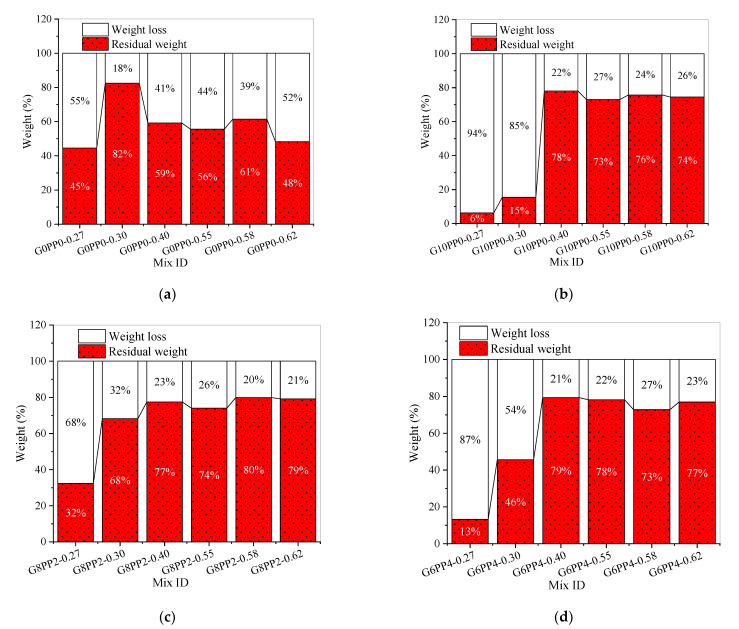
The weight loss and residual weight of mortar samples after wear abrasion test: (**a**) Wear abrasion test result of group 1; (**b**) Wear abrasion test result of group 2; (**c**) Wear abrasion test result of group 3; (**d**) Wear abrasion test result of group 4.

Wear resistance is an important parameter affecting the durability of cement-based materials [61]. Figure 4 shows the weight loss and residual weight of mortar samples after a wear abrasion test. It can be seen that the wear resistance of different samples varies greatly. In Figure 4a,b, the residual weight of mortar after the wear abrasion test is in good agreement with the trend of the compressive strength in Figure 2a,b. This is consistent with the discussion in reference [62]; that is, wear resistance increases with the increase in concrete compressive strength. In Figure 4a, the residual weight of G0PP0-0.30 in group 1 is 82%, and the weight loss is only 18%, while the weight loss of other samples in group 1 is close to or exceeds 40%. In Figure 4c, the residual weights of G8PP2-0.40, G8PP2-0.58 and G8PP2-0.62 were 77%, 80% and 79%, respectively. According to Figure 2c, Figure 3c and Figure 4c, the compressive strength of G8PP2-0.58 and G8PP2-0.62 is much lower than that of G8PP2-0.40, but the difference in the flexural strength of these samples is not so significant. The weight loss of 20% and 21% was lower than that of 23% of G8PP2-0.40; that is, in group 3, 1.6% GF and 0.4% PPF improved the wear resistance of G8PP2-0.58 and G8PP2-0.62 slightly better than the compressive strength and flexural strength. In Figure 4d, the wear resistance of G6PP4-0.40 is the best, with a residual weight of 79%. The compressive strength and flexural strength of G6PP4-0.55 and G6PP4-0.62 are much lower than those of G6PP4-0.40, but the gap is smaller compared with the wear resistance of G6PP4-0.40, and the residual weight reaches 78% and 77%, respectively. In references [53] and [54], the wear resistance of concrete containing fly ash at 91 and 270 days curing age was studied, respectively. Although the wear resistance has been improved in the above literature, the study on the wear resistance at 28 days conducted in this paper may be more valuable for engineering application.

At the lowest water/binder ratio (w/b = 0.27), the wear weight loss of G10PP0-0.27, G8PP2-0.27 and G6PP4-0.27 reached 94%, 68% and 87%; especially, G10PP0-0.27 and G6PP4-0.27 were worn into a crushed state. This is also the result of serious structural defects in mortar caused by the combined effect of low water/binder ratio, high volume mineral admixture and 2% volume fraction hybrid fiber.

**Figure 5:**
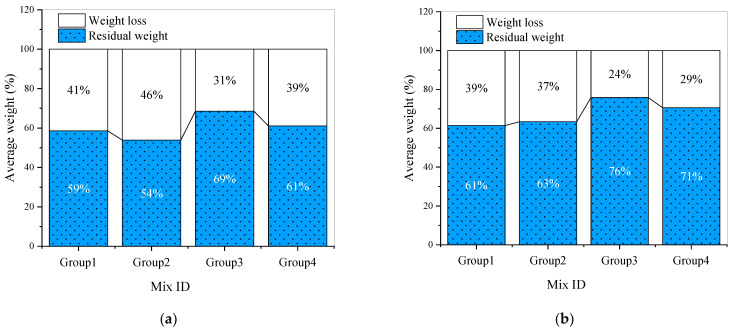
The average abrasion resistance level of each group of samples: (**a**) Average abrasion resistance level of each group of samples; (**b**) Average abrasion resistance level of each group of samples after removing the lowest water/binder ratio.

Figure 5a shows the average wear resistance of each group of samples. In order to eliminate the adverse effects of the lowest water/binder ratio on the wear abrasion test of samples, Figure 5b lists the average wear resistance of each group of samples after removing the 0.27 water/binder ratio. It can be seen that samples in group 3 have better performance on the wear abrasion test.

### 3.4. Microstructure

**Figure 6:**
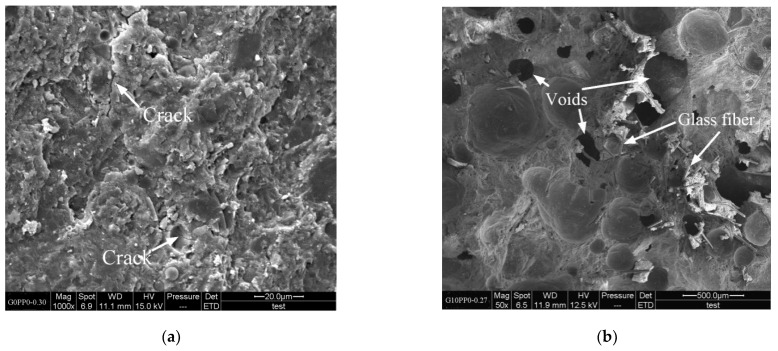

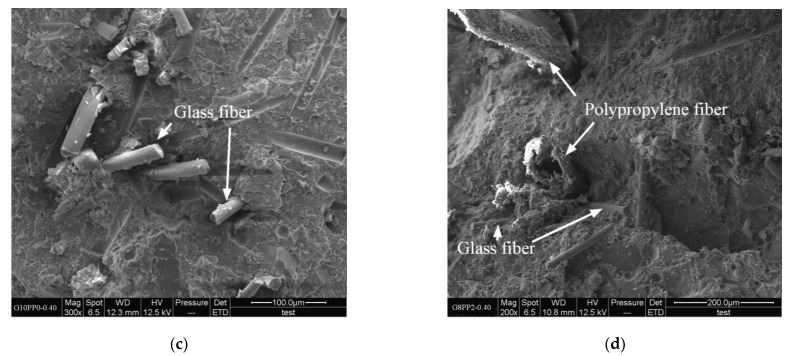
SEM images of mortars: (**a**) G0PP0-0.30; (**b**) G10PP0-0.27; (**c**) G10PP0-0.40; (**d**) G8PP2-0.40.

Figure 6 shows the scanning electron microscope (SEM) images of some representative mortar samples. Figure 6a shows the microstructure of G0PP0-0.30. It can be seen that when the fiber incorporation amount is 0, cracks occur on the gel surface due to hardening and shrinkage. As shown in Figure 6b, although no cracks were generated after 2% GF was added, the low water/binder ratio (w/b = 0.27) and fiber incorporation resulted in the poor working performance of fresh mortar and difficulty in molding, resulting in a large number of pores in mortar. In Figure 6c,d, it can be seen that the fibers are evenly distributed and the mortar structure is dense. In Figure 6d, GF and PPF form a three-dimensional staggered support network, and the cracks in the cementitious matrix can only continue to develop by bypassing the fiber, breaking the fiber, or pulling the fiber out, thus limiting the expansion of the crack tips in the cementitious matrix [33,57,63]. This is one of the important reasons for the improved strength and wear resistance of this mortar sample.

## 4. Conclusions

In this paper, the mechanical properties, wear resistance and microstructure of HFRM with high volume mineral admixtures (fly ash, silica fume and slag) were systematically studied under different water/binder ratios, and the following conclusions were obtained.

1. Comprehensively considering the compressive strength, flexural strength, wear resistance and microstructure of the mortar samples, G8PP2-0.40 is the optimal mix proportion. At this time, the replacement ratio of fly ash, silica fume and slag are: 20%, 5% and 30%, the water/binder ratio is 0.40, and the content of GF and PPF are 1.6% and 0.4%, respectively.
2. The incorporation of fibers has obvious negative effects on mortar samples with a low water/binder ratio (w/b = 0.27) and high content of mineral admixture.
3. Adding hybrid fiber could improve the wear resistance of mortar more obviously; the average residual weight of HFRM was higher after a wear abrasion test.

In this paper, the orthogonal experimental approach is used to carry out the mix ratio design; that is, by using the idea of mathematical statistics and the principle of orthogonality, representative points are selected from the full test points for the test. Although the orthogonal experimental approach can comprehensively consider multiple factors and greatly reduce the number of experiments, it also has certain limitations. For example, the optimal parameters obtained are only the combination of the parameters set in the experiment, and they may not be the globally optimal parameters. For the next step, according to the optimal parameters obtained in this paper, the range of variables in the test can be further reduced, and the full test can be conducted to find the global optimal parameters.

## Figures and Tables

**Table 1:** Physical and mechanical properties of cement.

Cement	Soundness	Specific Surface Area (m^2^/kg)	Setting Time (min)		Compressive Strength (MPa)		Flexural Strength(MPa)	
			Initial	Final	3 Days	28 Days	3 Days	28 Days
Experimental result	Qualified	324	159	223	23.6	48.9	6.3	8.1

**Table 2:** Chemical compositions of cement.

Chemical Compositions (%)	SiO_2_	CaO	Al_2_O_3_	Fe_2_O_3_	MgO	SO_3_	Na_2_O	K_2_O	Loss on Ignition
Cement	21.5	59.81	5.86	2.85	2.23	2.06	0.2	0.67	4.82

**Table 3:** Chemical compositions of fly ash.

Chemical Compositions (%)	SiO_2_	CaO	Al_2_O_3_	Fe_2_O_3_	MgO	SO_3_	Na_2_O	K_2_O	Loss on Ignition
Fly ash	66.67	3.05	18.97	4.39	1.24	0.3	-	-	5.38

**Table 4:** Chemical compositions of silica fume.

Chemical Compositions (%)	SiO_2_	CaO	Al_2_O_3_	Fe_2_O_3_	MgO	SO_3_	Na_2_O	K_2_O	Loss on Ignition
Silica fume	93.82	0.41	0.21	0	0.65	0.64	0.32	0.85	3.1

**Table 5:** Chemical compositions of slag.

Chemical Compositions (%)	SiO_2_	CaO	Al_2_O_3_	Fe_2_O_3_	MgO	SO_3_	Na_2_O	K_2_O	Loss on Ignition
Slag	32.08	38.09	15.06	0.94	8.26	0.17	-	-	5.4

**Table 6:** Properties of GF and PPF.

Type of Fiber	Length (mm)	Diameter (μm)	Aspect Ratio	Specific Gravity	Tensile Strength (MPa)	Elastic Modulus (MPa)
GF	12	15	800	2.36	1300	4286
PPF	12	60	200	0.91	486	4800

**Table 7:** Orthogonal factor level table.

Level	(A) Total Binder (kg/m3)	(B) Fly Ash (%)	(C) Silica Fume (%)	(D) Slag (%)	(E) w/b	(F) Fiber
1	300	0	0	15	0.27	0
2	350	20	5	30	0.3	2%GF + 0%PPF
3	400	30			0.4	1.6%GF + 0.4%PPF
4	450	40			0.55	1.2%GF + 0.8%PPF
5	480	50			0.58	
6					0.62	

**Table 8:** Mix proportions of mortar mixes.

Group	Mix ID	w/b	Total Binder (kg/m^3^)	Cement (kg/m^3^)	Fly Ash (kg/m^3^)	Silica Fume (kg/m^3^)	Slag (kg/m^3^)	Cement (%)	Fly Ash (%)	Silica Fume (%)	Slag (%)	GF (%)	PPF (%)
1	G0PP0-0.27	0.27	450	180	135	0	135	40	30	0	30	0	0
	G0PP0-0.30	0.3	400	160	160	20	60	40	40	5	15	0	0
	G0PP0-0.40	0.4	300	90	120	0	90	30	40	0	30	0	0
	G0PP0-0.55	0.55	450	67.5	225	22.5	135	15	50	5	30	0	0
	G0PP0-0.58	0.58	480	408	0	0	72	85	0	0	15	0	0
	G0PP0-0.62	0.62	350	140	105	0	105	40	30	0	30	0	0
2	G10PP0-0.27	0.27	350	157.5	140	0	52.5	45	40	0	15	2	0
	G10PP0-0.30	0.3	300	105	90	15	90	35	30	5	30	2	0
	G10PP0-0.40	0.4	400	220	120	0	60	55	30	0	15	2	0
	G10PP0-0.55	0.55	350	280	0	17.5	52.5	80	0	5	15	2	0
	G10PP0-0.58	0.58	400	80	200	0	120	20	50	0	30	2	0
	G10PP0-0.62	0.62	450	202.5	180	0	67.5	45	40	0	15	2	0
3	G8PP2-0.27	0.27	400	60	200	20	120	15	50	5	30	1.6	0.4
	G8PP2-0.30	0.3	450	292.5	90	0	67.5	65	20	0	15	1.6	0.4
	G8PP2-0.40	0.4	350	157.5	70	17.5	105	45	20	5	30	1.6	0.4
	G8PP2-0.55	0.55	480	192	144	0	144	40	30	0	30	1.6	0.4
	G8PP2-0.58	0.58	450	180	180	22.5	67.5	40	40	5	15	1.6	0.4
	G8PP2-0.62	0.62	400	260	0	20	120	65	0	5	30	1.6	0.4
4	G6PP4-0.27	0.27	300	180	60	15	45	60	20	5	15	1.2	0.8
	G6PP4-0.30	0.3	350	70	175	0	105	20	50	0	30	1.2	0.8
	G6PP4-0.40	0.4	450	135	225	22.5	67.5	30	50	5	15	1.2	0.8
	G6PP4-0.55	0.55	400	180	160	0	60	45	40	0	15	1.2	0.8
	G6PP4-0.58	0.58	350	122.5	105	17.5	105	35	30	5	30	1.2	0.8
	G6PP4-0.62	0.62	480	144	240	24	72	30	50	5	15	1.2	0.8

## Data Availability

No new data were created or analyzed in this study. Data sharing is not applicable to this article.
